# Antagonizing MDM2 Overexpression Induced by MDM4 Inhibitor CEP-1347 Effectively Reactivates Wild-Type p53 in Malignant Brain Tumor Cells

**DOI:** 10.3390/cancers15174326

**Published:** 2023-08-30

**Authors:** Yuta Mitobe, Shuhei Suzuki, Yurika Nakagawa-Saito, Keita Togashi, Asuka Sugai, Yukihiko Sonoda, Chifumi Kitanaka, Masashi Okada

**Affiliations:** 1 Department of Molecular Cancer Science, School of Medicine, Yamagata University, 2-2-2 Iida-nishi, Yamagata 990-9585, Japan; 2 Department of Neurosurgery, School of Medicine, Yamagata University, 2-2-2 Iida-nishi, Yamagata 990-9585, Japan; 3 Department of Clinical Oncology, School of Medicine, Yamagata University, 2-2-2 Iida-nishi, Yamagata 990-9585, Japan; 4 Department of Ophthalmology and Visual Sciences, School of Medicine, Yamagata University, 2-2-2 Iida-nishi, Yamagata 990-9585, Japan; 5 Research Institute for Promotion of Medical Sciences, Faculty of Medicine, Yamagata University, 2-2-2 Iida-nishi, Yamagata 990-9585, Japan

**Keywords:** MDMX, HDMX, HDM4, MDM2 antagonist, drug repositioning, drug repurposing, feedback mechanism, combination therapy, meningioma, glioma

## Abstract

**Simple Summary:**

In human cancer, the major tumor suppressor p53 is often functionally inactivated through the deregulation of its negative regulators MDM2 and MDM4. The inhibitors of MDM4 have recently been attracting increasing attention as a promising approach to the reactivation of wild-type p53 in cancer cells. However, due to the multiple, complex feedback loops that involve p53, MDM2, and MDM4, the mechanisms by which the inhibition of MDM4 affects MDM2 and whether the additional inhibition of MDM2 enhances the p53-activating effects of MDM4 inhibitors remain unclear. Here, we demonstrated that CEP-1347, an inhibitor of MDM4 protein expression, induced the overexpression of MDM2 and that the concomitant inhibition of MDM2, particularly the selective disruption of the MDM2–p53 interaction, promoted the CEP-1347-mediated activation of p53 and the inhibition of cell growth. The present results suggest the potential of combining the inhibition of MDM4 with the disruption of the MDM2–p53 interaction to maximally activate p53 in cancer cells.

**Abstract:**

The development of MDM4 inhibitors as an approach to reactivating p53 in human cancer is attracting increasing attention; however, whether they affect the function of MDM2 and how they interact with MDM2 inhibitors remain unknown. We addressed this question in the present study using CEP-1347, an inhibitor of MDM4 protein expression. The effects of CEP-1347, the genetic and/or pharmacological inhibition of MDM2, and their combination on the p53 pathway in malignant brain tumor cell lines expressing wild-type p53 were investigated by RT-PCR and Western blot analyses. The growth inhibitory effects of CEP-1347 alone or in combination with MDM2 on inhibition were examined by dye exclusion and/or colony formation assays. The treatment of malignant brain tumor cell lines with CEP-1347 markedly increased MDM2 protein expression, while blocking CEP-1347-induced MDM2 overexpression by genetic knockdown augmented the effects of CEP-1347 on the p53 pathway and cell growth. Blocking the MDM2–p53 interaction using the small molecule MDM2 inhibitor RG7112, but not MDM2 knockdown, reduced MDM4 expression. Consequently, RG7112 effectively cooperated with CEP-1347 to reduce MDM4 expression, activate the p53 pathway, and inhibit cell growth. The present results suggest the combination of CEP-1347-induced MDM2 overexpression with the selective inhibition of MDM2′s interaction with p53, while preserving its ability to inhibit MDM4 expression, as a novel and rational strategy to effectively reactivate p53 in wild-type p53 cancer cells.

## 1. Introduction

The tumor suppressor gene *TP53* encodes a transcription factor, the p53 protein, which elicits cellular responses, such as cell cycle arrest and apoptosis, through the transcriptional activation of its target genes in response to genotoxic stresses, which may cause genetic aberrations that culminate in the neoplastic transformation of cells. Therefore, the p53 protein plays a key role in maintaining the integrity of the genome and in the prevention of carcinogenesis, as evidenced by p53 being inactivated, either genetically or functionally, in virtually all human cancers [1,2,3,4,5]. In the control of p53 activity, the oncoproteins MDM2 and MDM4 are recognized as major negative regulators because of their ability to directly interact with p53 and thereby inhibit its transcriptional activity and stability, alone or in complex with each other [6,7]. The deregulation of MDM2 and MDM4 has been shown to play a pivotal role in the functional inactivation of p53; therefore, they are attractive molecular targets for the therapeutic reactivation of p53 in cancer cells retaining wild-type p53. Accordingly, inhibitors of MDM2 and MDM4 are currently at various stages of preclinical and clinical development [5,6,7,8,9,10].

CEP-1347 is a small molecule inhibitor of mixed lineage kinases which we recently identified as a novel inhibitor of MDM4. CEP-1347 reduces the protein, but not the mRNA, expression of MDM4, activates the p53 pathway, and effectively inhibits the growth of cancer cells expressing MDM4 and wild-type p53; however, the mechanisms underlying the CEP-1347-mediated inhibition of MDM4 remain unclear [11,12,13]. A clear advantage of CEP-1347 over other inhibitors of MDM2 and/or MDM4 that have been developed to date is that the long-term safety of CEP-1347 in humans has been demonstrated in a clinical trial, and it inhibits MDM4 at clinically relevant concentrations in vitro [11,12,13,14]. Most importantly, we recently reported using a preclinical mouse model of meningioma; the systemic administration of CEP-1347, at a dose that was 1/10 the mouse equivalent of the dose safely administered to humans, was sufficient to effectively inhibit the growth of meningioma xenografts [13], suggesting the potential of CEP-1347 as a candidate wild-type p53-targeting agent for clinical applications. In the course of our research, we found that the MDM4 inhibition-mediated activation of p53 by CEP-1347 was accompanied by a marked increase in the expression of MDM2, a representative transcriptional target of p53. However, since the ability of MDM2 to destabilize p53 was previously shown to be dependent on MDM4 [15,16,17], it remains unknown whether and to what extent the CEP-1347-induced up-regulation of MDM2 counteracts the activation of p53 when the MDM4 levels are low as a result of CEP-1347-mediated inhibition. Consequently, it has not yet been established whether the concomitant inhibition of MDM2 further enhances the CEP-1347-mediated activation of p53. In the present study, we addressed these questions and the results obtained demonstrated that the inhibition of MDM2 contributed to the efficient activation of p53 by CEP-1347.

## 2. Materials and Methods

### 2.1. Reagents and Antibodies

CEP-1347 was purchased from TOCRIS Bioscience (Bristol, UK) and dissolved in DMSO to prepare a 0.5 mM stock solution. RG7112 was purchased from Selleck Chemicals (Houston, TX, USA) and dissolved in DMSO to prepare a 5 mM stock solution. Trypan blue solution (T8154) was obtained from Merck KGaA (Darmstadt, Germany). An antibody against murine double minute 4 (MDM4, A700-000-T) was purchased from BETHYL (FORTIS LIFE SCIENCES, Waltham, MA, USA); an antibody against MDM2 (AF1244) was from R&D Systems (Minneapolis, MN, USA); the antibodies against cyclin-dependent kinase inhibitor 1A (CDKN1A, p21^Waf1/Cip1^) (#2947) and GAPDH (#5174) were from Cell Signaling Technology, Inc. (Beverly, MA, USA); and an antibody against p53 (sc-126) was from Santa Cruz Biotechnology, Inc. (Santa Cruz, CA, USA).

### 2.2. Cell Culture

IOMM-Lee, a human malignant meningioma cell line, and IMR90, normal human fetal lung fibroblasts, were obtained from the American Type Culture Collection (Manassas, VA, USA) and cultured in DMEM supplemented with 5% or 10% fetal bovine serum (FBS), respectively. The human glioblastoma cell lines, A172 and T98G, were provided by the RIKEN RBC (Tsukuba, Japan) through the National BioResource Project of the Ministry of Education, Culture, Sports, Science and Technology, Japan and cultured in RPMI 1640 medium supplemented with 10% FBS. The culture media were supplemented with 100 U/mL penicillin and 100 μg/mL streptomycin. All the IMR90 experiments were performed using cells with a low passage number (<8).

### 2.3. Western Blot Analysis

A Western blot analysis was conducted as previously described [18,19]. The cells were harvested and washed with ice-cold phosphate-buffered saline (PBS) and then lysed in RIPA buffer (10 mM Tris/HCl [pH 7.4], 0.1% sodium dodecyl sulfate (SDS), 1% Nonidet P-40, 0.1% sodium deoxycholate, 150 mM NaCl, 1 mM EDTA, 1.5 mM sodium orthovanadate, 10 mM sodium pyrophosphate, 10 mM sodium fluoride, and protease inhibitor cocktail set III (FUJIFILM Wako Chemicals, Osaka, Japan)). Lysates were immediately mixed with the same volume of 2 × Laemmli buffer (125 mM Tris/HCl [pH 6.8], 4% SDS, and 10% glycerol) and boiled at 95 °C for 10 min. After the protein concentrations of the cell lysates were measured using a BCA protein assay kit (Thermo Fisher Scientific, Waltham, MA, USA), the samples containing equal amounts of protein were separated by SDS-polyacrylamide gel electrophoresis and transferred to polyvinylidene difluoride membranes. The membranes were probed with the indicated primary antibodies followed by appropriate horseradish peroxidase (HRP)-conjugated secondary antibodies as recommended by the manufacturer of each antibody. Regarding reprobing, the antibodies were stripped from the probed membranes using stripping buffer (2% SDS, 100 mM β-mercaptoethanol, and 62.5 mM Tris-HCl (pH 6.8)). After stripping, the membranes were washed with Tris-buffered saline with Tween 20 and blocked with skim milk. The membranes were then reprobed with the appropriate antibodies. Immunoreactive bands were visualized using Immobilon Western Chemiluminescent HRP Substrate (Merck KGaA) and detected by a ChemiDoc Touch device (Bio-Rad, Hercules, CA, USA).

### 2.4. Reverse Transcription (RT)-PCR Analysis

An RT-PCR analysis was conducted as previously described [12]. Total RNA was extracted from the cells using Trizol (Thermo Fisher Scientific), and 1 μg of total RNA was reverse transcribed using the PrimeScript II 1st strand cDNA Synthesis kit (Takara Bio Inc., Shiga, Japan) according to the manufacturer’s protocol. Target genes were amplified with Quick Taq HS DyeMix (Toyobo Co., Ltd., Osaka, Japan). The sequences of gene-specific PCR primer sets listed in Table 1 were designed using Primer-BLAST (https://www.ncbi.nlm.nih.gov/tools/primer-blast/ (accessed on 10 May 2019)).

### 2.5. Gene Silencing by siRNA

siRNAs against human MDM2 (#1: HSS142909, #2: HSS142910, and #3: HSS142911) and Stealth RNAi™ siRNA Negative Control Med GC Duplex #2 were purchased from Thermo Fisher Scientific. The cells were transiently transfected with one of the siRNAs against MDM2 (siMDM2; 160–200 pmol per 6 cm dish) or with control siRNA (siCt; 160–200 pmol per 6 cm dish) using Lipofectamine RNAiMAX (Thermo Fisher Scientific) according to the manufacturer’s instructions.

### 2.6. Trypan Blue Dye Exclusion Assay

The numbers of viable cells were measured using the trypan blue dye exclusion assay [12]. Adherent and non-adherent cells in culture dishes were collected, centrifuged, resuspended in PBS, and stained with 0.2% trypan blue for 1 min. Viable cells were identified by their ability to exclude trypan blue using a hemocytometer.

### 2.7. Colony Formation Assay

A colony formation assay was performed as previously described [13]. In brief, IOMM-Lee cells were seeded at a low, colony-forming density (200 cells/12-well plate). After being treated and cultured as described in the figure legend, the cells were fixed with paraformaldehyde (4% *w*/*v*), followed by staining with crystal violet (0.1% *w*/*v*). The colonies (consisting of ≥50 cells derived from a single cell) were counted using a microscope.

### 2.8. Statistical Analysis

The results are shown as means + standard deviations (SD). The data were analyzed using the Student’s *t*-test for comparisons between the two groups. Differences with a *p*-value < 0.05 were considered to be significant.

## 3. Results

### 3.1. Marked Increase in the Expression of MDM2 after the CEP-1347 Treatment of Malignant Brain Tumor Cells with Wild-Type p53

**Figure 1:**
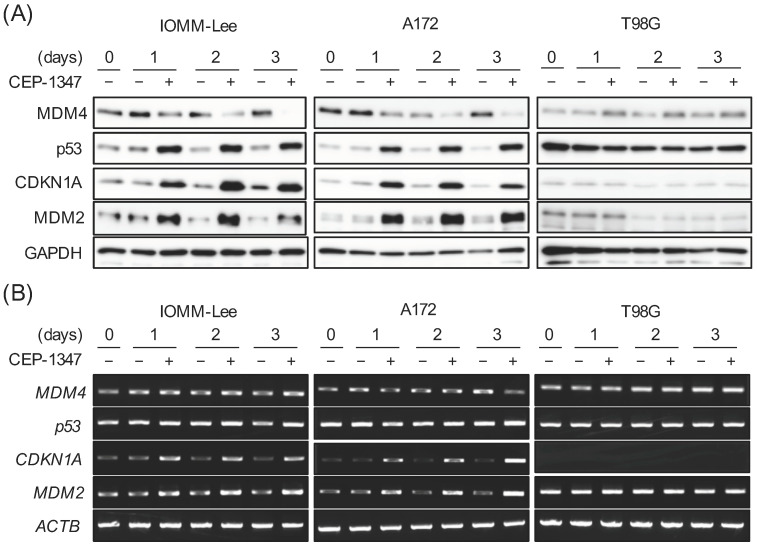
The CEP-1347 treatment markedly increases MDM2 in malignant brain tumor cells with wild-type p53. IOMM-Lee, A172, and T98G cells treated without or with 250 nM CEP-1347 for the indicated time periods were subjected to Western blot (**A**) and RT-PCR (**B**) analyses for the expression of murine double minute (MDM) 4, p53, cyclin-dependent kinase inhibitor 1A (CDKN1A, p21^Waf1/Cip1^), and MDM2. Similar results were obtained from more than two independent biological replicates. Original blot images can be found in Figure S1.

We previously reported that CEP-1347 inhibited the expression of MDM4 and activated the p53 pathway in retinoblastoma, glioma, and meningioma cell lines expressing wild-type p53 [11,12,13]. As the kinetics of the expression of MDM4, p53, and p53 target gene products after the CEP-1347 treatment have not yet been examined in brain tumor cell lines (i.e., glioma and meningioma cell lines), we conducted a time course analysis of their expression in wild-type p53-expressing glioblastoma (A172) and meningioma (IOMM-Lee) cell lines. In agreement with our previous results, the CEP-1347 treatment decreased and increased the protein levels of MDM4 and p53, respectively, and induced the mRNA and protein expression of the p53 target genes *CDKN1A* and *MDM2*. In contrast, CEP-1347 neither reduced the expression of MDM4 nor increased the expression of p53 and its target gene products in T98G, a glioblastoma cell line expressing a mutant p53; this was in line with our previous finding that CEP-1347 inhibits MDM4 expression and the p53 pathway specifically in wild-type p53 glioma cells [12]. Notably, we observed a marked increase in MDM2 protein expression as early as 1 day after the CEP-1347 treatment in IOMM-Lee and A172 cells (Figure 1A,B), in which we previously showed that the MDM2 protein was more highly expressed than its mRNA under unstimulated culture conditions [12,13]. Intriguingly, whereas the reduction in MDM4 protein levels was still partial 1 day after the CEP-1347 treatment and progressed thereafter, the p53 protein levels, together with the mRNA and protein levels of CDKN1A/p21 and MDM2, nearly reached their peaks 1 day after the CEP-1347 treatment and remained constant thereafter (Figure 1A,B), suggesting that the early increase in MDM2 expression interfered with the activation of p53 by CEP-1347 at later time points.

### 3.2. CEP-1347-Induced MDM2 Overexpression Counteracts the CEP-1347-Induced Activation of p53

**Figure 2:**
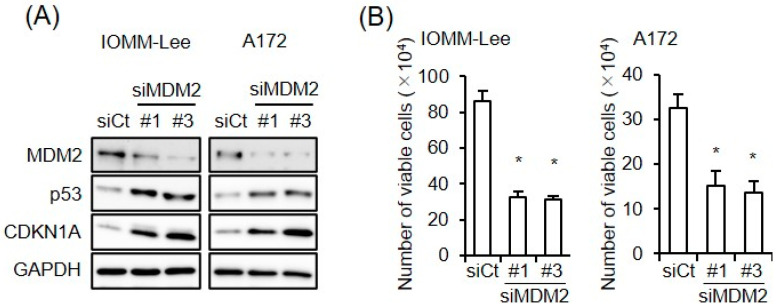
Endogenous expression of MDM2 contributes to p53 inactivation. (**A**) IOMM-Lee and A172 cells were transiently transfected with the indicated siRNA against MDM2 (#1 and #3) or with control siRNA (siCt). After being cultured for 3 days, cells were subjected to a Western blot analysis. (**B**) Cells were transiently transfected as in (**A**). After 3 days, transfected cells were subjected to the trypan blue dye exclusion assay to measure the number of viable cells. Values represent means + SD from triplicate samples of a representative experiment. * *p* < 0.05 vs. siCt-transfected cells. Similar results were obtained from more than two independent biological replicates. Original blot images can be found in Figure S2.

**Figure 3:**
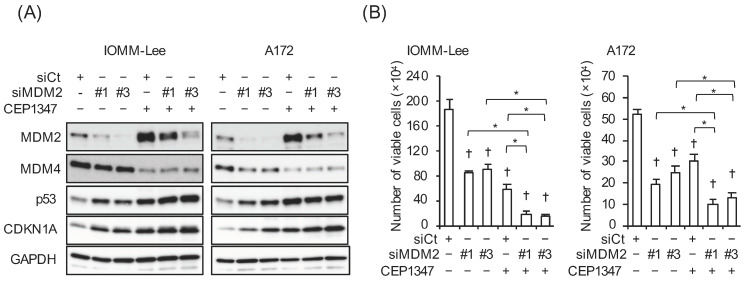
CEP-1347-induced MDM2 overexpression counteracts the CEP-1347-induced activation of p53. IOMM-Lee and A172 cells were transiently transfected with the indicated siRNA against MDM2 (#1 and #3) or with control siRNA (siCt). After being cultured for 1 day, cells treated without or with 250 nM CEP-1347 for 2 and 3 additional days were subjected to a Western blot analysis (**A**) and the trypan blue dye exclusion assay (**B**), respectively. Values represent the means + SD of triplicate samples of a representative experiment. * *p* < 0.05. † *p* < 0.05 vs. siCt-transfected cells treated without CEP-1347. Similar results were obtained from more than three independent biological replicates. Original blot images can be found in Figure S3.

With the aim of examining the impact of the increased expression of MDM2 on the CEP-1347-induced activation of p53, we conducted a series of pilot experiments, in which we initially tested three siRNAs targeting different regions of MDM2 for their efficiency in knocking down MDM2 in IOMM-Lee and A172 cells. As two of the siRNAs (#1 and #3) clearly knocked down MDM2 (Figure 2A), they were used in the subsequent experiments. Of note, the knockdown of MDM2 increased the expression of p21/CDKN1A in parallel with p53 and inhibited the growth of IOMM-Lee and A172 cells (Figure 2A,B), suggesting that both of these cell lines expressed functional MDM2 and p53 and also that the endogenous expression of MDM2 contributed to the inactivation of p53 in these cell lines. We then investigated whether the increase in MDM2 expression induced by the CEP-1347 treatment attenuated the effects of CEP-1347 on p53 expression and activity. In both cell lines, the knockdown of MDM2 in combination with the CEP-1347 treatment increased p53 activity, as represented by the higher expression of p53 and p21/CDKN1A compared with that of the CEP-1347 treatment alone, whereas only slight changes in p53 levels were observed in A172 cells (Figure 3A). In accordance with the increase in p53 activity induced by the knockdown of MDM2, the growth inhibitory effects of CEP-1347 were also enhanced (Figure 3B). These results suggest that the increased expression of MDM2 induced by CEP-1347 played a significant role in attenuating the p53-activating effects of CEP-1347; this prompted us to examine whether the concomitant targeting of MDM2 with an MDM2 inhibitor enhances the therapeutic effects of CEP-1347.

### 3.3. The MDM2 Antagonist RG7112 Concomitant with CEP-1347 Effectively Activates p53 and Inhibits the Growth of Malignant Brain Tumor Cells with Wild-Type p53

**Figure 4:**
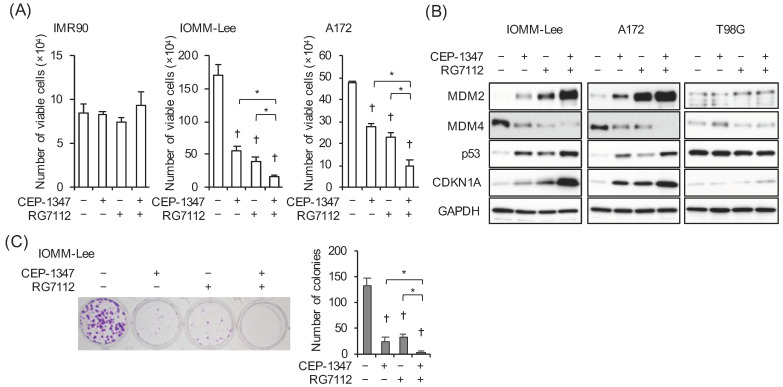
The MDM2 antagonist RG7112 concomitant with CEP-1347 effectively activates p53 and inhibits the growth of malignant brain tumor cells expressing wild-type p53. (**A**) IMR90 normal human fibroblasts, IOMM-Lee cells, and A172 cells treated with the indicated drugs (CEP-1347, 250 nM; RG7112, 500 nM) for 3 days were subjected to the trypan blue dye exclusion assay. (**B**) IOMM-Lee, A172, and T98G cells treated with the indicated drugs (CEP-1347, 250 nM; RG7112, 500 nM) for 2 days were subjected to a Western blot analysis. (**C**) Cells treated with the indicated drugs (CEP-1347, 250 nM; RG7112, 500 nM) for 3 days were cultured for another 5 days in the absence of any drugs for the colony formation assay. Representative images (left panels) and the number of colonies (right graphs) are shown. Values represent means + SD from triplicate samples of a representative experiment. * *p* < 0.05. † *p* < 0.05 vs. cells treated without any drugs. Similar results were obtained from more than two independent biological replicates. Original blot images can be found in Figure S4.

To investigate the impact of the pharmacological inhibition of MDM2 in a therapeutically relevant setting, we selected RG7112, a pharmacological inhibitor of the MDM2–p53 interaction with demonstrated efficacy in preclinical models of glioblastoma as well as the ability to penetrate the blood–brain barrier, for which clinical information regarding safety is available [20,21,22]. We then selected the concentration of RG7112 that did not impair the growth of normal cells in the presence of CEP-1347. After confirming that RG7112 at 500 nM did not inhibit the growth of IMR90 normal human fibroblasts alone or in combination with 250 nM CEP-1347 (Figure 4A), we treated IOMM-Lee and A172 cells with these concentrations of CEP-1347 and RG7112 alone or in combination to investigate the mechanisms by which RG7112 modulated the CEP-1347-mediated activation of the p53 pathway. In agreement with the results of the MDM2 knockdown experiments, the combination of CEP-1347 and RG7112 induced the expression of p21/CDKN1A and MDM2 as well as that of p53 more efficiently than either of them alone (Figure 4B), which further corroborated the CEP-1347-induced overexpression of MDM2 exerting inhibitory effects on the CEP-1347-induced activation of p53. It is important to note that the MDM4 protein levels were lower in the presence of RG7112 (Figure 4B), which is consistent with previous findings [23,24], and RG7112 further reduced MDM4 expression in combination with CEP-1347, which indicated that RG7112 effectively cooperated with CEP-1347 to inhibit MDM4 expression. The combinatorial action of RG7112 and CEP-1347 was apparently specific to the wild-type p53 cells, given the lack of effects of the combination on the p53-mutant T98G cells (Figure 4B). We next investigated whether the efficient induction of the CDK inhibitor p21/CDKN1A in A172 and IOMM-Lee cells by the drug combination translated into efficient growth inhibition. The results obtained indicated that, in parallel with the expression levels of p21, the combination of CEP-1347 and RG7112 inhibited the growth of malignant brain tumor cells more effectively than either of them alone (Figure 4A). As IOMM-Lee cells form colonies when seeded at low densities, we also conducted a colony formation assay to assess the impact of CEP-1347 and RG7112 on the clonogenic survival of IOMM-Lee cells. Although colony formation was significantly inhibited, colonies still formed when the cells were treated with either of the drugs alone. However, colony formation was almost abolished when IOMM-Lee cells were treated with the combination of CEP-1347 and RG7112 (Figure 4C), underscoring the therapeutic potential of this drug combination.

## 4. Discussion

MDM2 and MDM4 are two major negative regulators of p53 that act on p53 either alone or in complex with each other, inhibiting the transcriptional activity of p53 independently of each other and forming a heterodimer to efficiently destabilize p53 through ubiquitination. Despite being homologous proteins, MDM2 and MDM4 are clearly distinct in that only MDM2 exhibits E3 ligase activity to promote the degradation of MDM4 and MDM2 itself, in addition to p53. The relationship between MDM2 and MDM4 is reciprocal; MDM2 promotes the ubiquitination of MDM4, while MDM4 inhibits the self-ubiquitination of MDM2. To further complicate the tripartite relationship, the *MDM2* gene is a direct transcriptional target of p53 [7,10]. The complex interplay between p53, MDM2, and MDM4 makes it almost impossible to predict the net effects of MDM4 inhibition on MDM2 expression. In our previous study on the effects of CEP-1347 on retinoblastoma cells, we showed that the CEP-1347-mediated inhibition of MDM4 expression increased the expression of MDM2 in one cell line but decreased that of MDM2 in another [11]. In the present study, we conducted a time course analysis of the expression of molecules along the p53 pathway after the treatment of malignant brain tumor cells with CEP-1347, which revealed a marked increase in MDM2 expression, particularly in its protein levels, induced by CEP-1347. The mechanisms underlying the marked increase in MDM2 protein levels currently remain unclear. However, since the malignant brain tumor cell lines used in the present study were characterized by the higher expression of the MDM2 protein than its mRNA, some post-transcriptional mechanisms involved therein may also be involved in the highly efficient induction of MDM2 protein expression by CEP-1347. The result showing the marked increase in MDM2 expression after the CEP-1347 treatment led to the next question, which is also the primary question, of the present study: whether and to what extent increases in MDM2 expression affected the CEP-1347-induced activation of p53 and, consequently, the inhibition of cell growth. Although MDM2 has been shown to promote the ubiquitination and degradation of p53 as a homodimer, it exerts these effects more efficiently as a heterodimer with MDM4 [15,16,17]. Therefore, the efficiency of increased MDM2 in destabilizing p53 when the MDM4 expression was reduced by CEP-1347 remains unclear. We addressed this question in the present study and demonstrated, using both genetic and pharmacological approaches, that the inhibition of MDM2 effectively promoted CEP-1347-induced increases in p53 protein levels and activity and its inhibition of cell growth. It might be important to note here that, while the expression levels of p21/CDKN1A, the major transcriptional target of p53, were invariably increased when MDM2 was inhibited in the presence of CEP-1347, either genetically or pharmacologically, only slight changes were noted in the p53 protein levels when MDM2 was knocked down in A172 cells (Figure 3A). This result provides evidence that the increased MDM2 expression contributes to the inactivation of the p53 pathway not only by promoting the destabilization of p53 but also by inhibiting its transcriptional activity.

Although the development of MDM4 inhibitors lagged behind that of MDM2 inhibitors, it has been increasingly promoted in recent years, in part by research showing that the persistent expression of MDM4 in cancer cells counteracted the cytotoxic effects of MDM2 inhibition [7,10,24]. MDM4 inhibitors are mainly divided into two categories based on the strategy adopted to target MDM4: one is by directly blocking the MDM4–p53 interaction and the other is by reducing the MDM4 protein levels through the inhibition of MDM4 expression or the promotion of MDM4 degradation [10]. CEP-1347 belongs to the latter category of “MDM4 protein reducers”; however, limited information is currently available on the combination of this type of MDM4 inhibitor with MDM2 inhibitors. Although a previous study showed that 17AAG, an Hsp90 inhibitor, promoted the degradation of MDM4 and synergized with nutlin, a prototype member of the MDM2 inhibitors, to induce p53-mediated apoptosis in solid tumors; whether the synergy between 17AAG and nutlin was due to the ability of 17AAG to reduce MDM4 expression was not elucidated [25]. Another study identified the small molecule XI-006 as a repressor of MDM4 transcription and demonstrated that XI-006 induced apoptosis by inhibiting MDM4 expression and activating p53. This study also showed that XI-006 enhanced both the p53-activating and the anticancer effects of nutlin-3a [26]. However, the impact of the XI-006 treatment on MDM2 expression was not examined, and the mechanisms underlying the combined effects of XI-006 and nutlin-3a remain unknown. In conjunction with these findings, the present results not only reinforce the combination of “MDM4 protein reducers” with MDM2 inhibitors as a viable approach to treating cancer cells with wild-type p53, but further provide the first insight into the mechanisms underlying these effects.

In the present study, the inhibition of MDM2 by RG7112 reduced the protein levels of MDM4 (Figure 4B). In contrast, the knockdown-mediated inhibition of MDM2 did not affect the MDM4 protein levels, with the exception of the MDM2 knockdown in the absence of CEP-1347 in A172 cells (Figure 3A). The disparate effects of these two different methods of MDM2 inhibition on MDM4 protein expression may be explained by the positive feedback loop formed by p53, MDM2, and MDM4; activated p53 promotes the transcription of MDM2, and increased MDM2 then promotes the degradation of MDM4, culminating in the further activation of p53 [23]. Given this positive feedback loop, it is highly conceivable that the knockdown of MDM2 may disrupt this loop, whereas RG7112 may reinforce it by alleviating the negative impact of increased MDM2 on p53. Although the reduced expression of MDM4 upon the knockdown of MDM2 alone in A172 cells (Figure 3A) does not appear to be explained based on literature findings, the present results as a whole are consistent with this idea and suggest that blocking the interaction between p53 and MDM2 without inhibiting MDM2 expression is an effective approach to reinforcing the positive feedback loop. Importantly, since the overexpression of MDM4 is a factor that may interfere with this feedback loop, a reduction in the expression of MDM4 is also regarded as an effective approach. Therefore, the combination of CEP-1347 with RG7112 is a rational strategy not only because RG7112 blocks the negative impact of increased MDM2 expression on p53 after the CEP-1347 treatment, but also because CEP-1347-mediated reductions in the expression of MDM4 facilitate the positive feedback loop of p53 activation driven by RG7112. The present results showing that CEP-1347 in combination with RG7112 effectively activated the p53 pathway and inhibited the growth of malignant brain tumor cells corroborate the theoretical superiority of this combination, which warrants further study in clinical settings.

To ascertain the clinical relevance of the results obtained herein, we used CEP-1347 at 250 nM, a concentration that is markedly lower than the maximal plasma concentration (~700 nM) in humans [27], throughout the experiments conducted in the present study. Furthermore, in consideration of its potential dose-limiting toxicities, such as thrombocytopenia [22,28], we used RG7112 at very low concentrations, i.e., up to 500 nM, when its plasma concentration may reach well above 5 μM in humans after its oral administration [22,29]. Regarding RG7112, a previous study showed that RG7112 at 5 μM significantly inhibited platelet formation and induced apoptosis in megakaryocytes in vitro, but it also showed that negative effects were minimal when it was used at 1 μM [30]. Furthermore, our preliminary data showed that RG7112, at concentrations between 500 nM and 1 μM, significantly inhibited the growth of IMR90 normal fibroblasts. Therefore, we used RG7112 at or below 500 nM in the present study, and the results obtained clearly demonstrated that the combination of 500 nM RG7112 with 250 nM CEP-1347 inhibited the growth of malignant brain tumor cells more effectively than either of them alone without exerting growth inhibitory effects on the IMR90 cells. Although the safety and efficacy of combining CEP-1347 with RG7112 or other MDM2 inhibitors need to be investigated in future preclinical and clinical studies, our in vitro results suggest that this combination represents a feasible and effective approach in the treatment of malignant brain tumors with wild-type p53.

## 5. Conclusions

Here, we demonstrated that the MDM4 inhibitor CEP-1347 markedly increased MDM2 expression in malignant brain tumor cells, which, in turn, interfered with the CEP-1347-induced activation of p53, which may be effectively prevented by blocking the MDM2–p53 interaction. The present results, by providing a mechanistic rationale for the combination, indicate that the combination of the inhibitors of MDM4 expression with those targeting the p53–MDM2 interaction is a rational strategy to efficiently reactivate the p53 pathway in wild-type p53 cancer cells. As the safety profile of CEP-1347 is known in humans, CEP-1347 combined with MDM2 inhibitors in clinical development represents an attractive approach to treating cancers with wild-type p53.

## Figures and Tables

**Table 1:** Sequences of PCR primers.

Gene Name	Forward	Reverse
*MDM4*	AGGTACGACCAAAACTGCCG	CTGCACTTTGCTTCAGTTGGT
*MDM2*	GGTGCTGTAACCACCTCACA	TGAGTCCGATGATTCCTGCTG
*TP53*	ACAACGTTCTGTCCCCCTTG	CTCCGTCATGTGCTGTGACT
*CDKN1A*	GGGATTTCTTCTGTTCAGGCG	TGGTAGAAATCTGTCATGCTGGT
*ACTB*	CCCATGCCATCCTGCGTCTG	CGTCATACTCCTGCTTGCTG

## Data Availability

Data are contained within the article.

## Supplementary Materials

The following supporting information can be downloaded at: https://www.mdpi.com/article/10.3390/cancers15174326/s1, Figure S1: Uncropped Western blot and RT-PCR images for Figure 1, Figure S2: Uncropped Western blot images for Figure 2, Figure S3: Uncropped Western blot images for Figure 3, Figure S4: Uncropped Western blot images for Figure 4.
