# Supplementary material for: Antagonizing MDM2 Overexpression Induced by MDM4 Inhibitor CEP-1347 Effectively Reactivates Wild-Type p53 in Malignant Brain Tumor Cells
Source: Cancers (Basel). 2023 Aug 30;15(17):4326. doi: 10.3390/cancers15174326 (PMC10486490; doi:10.3390/cancers15174326)
Supplement: Supplementary file 1 [file cancers-15-04326-s001.zip › cancers-2525265-supplementary.pdf]

Supplementary Materials: Antagonizing MDM2 Overexpression Induced by MDM4 Inhibitor CEP-1347 Effectively Reactivates Wild-Type p53 in Malignant Brain Tumor Cells

Yuta Mitobe, Shuhei Suzuki, Yurika Nakagawa-Saito, Keita Togashi, Asuka Sugai, Yukihiko Sonoda, Chifumi Kitanaka and Masashi Okada

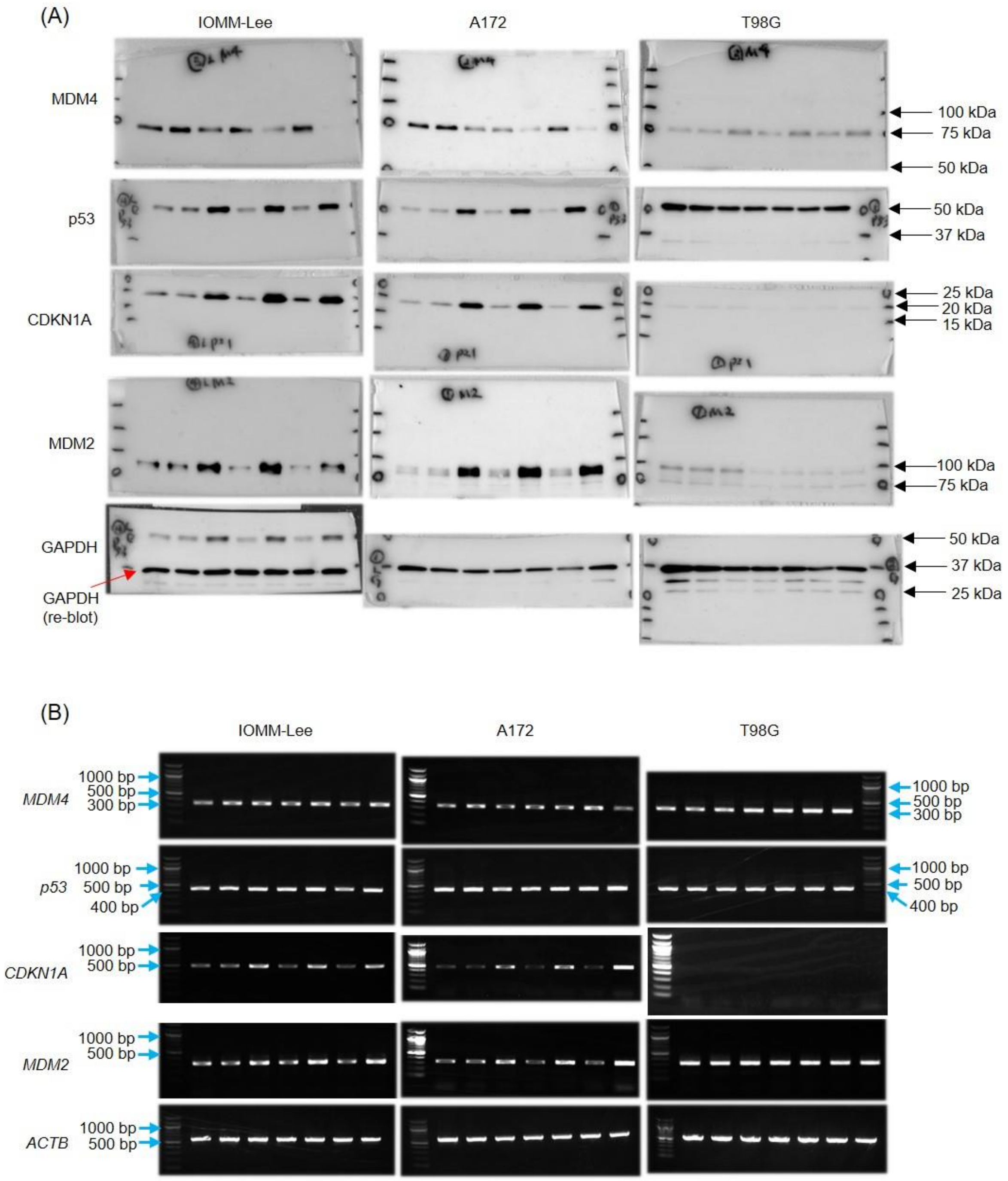

Figure S1. Uncropped Western Blot and RT-PCR images for Figure 1.

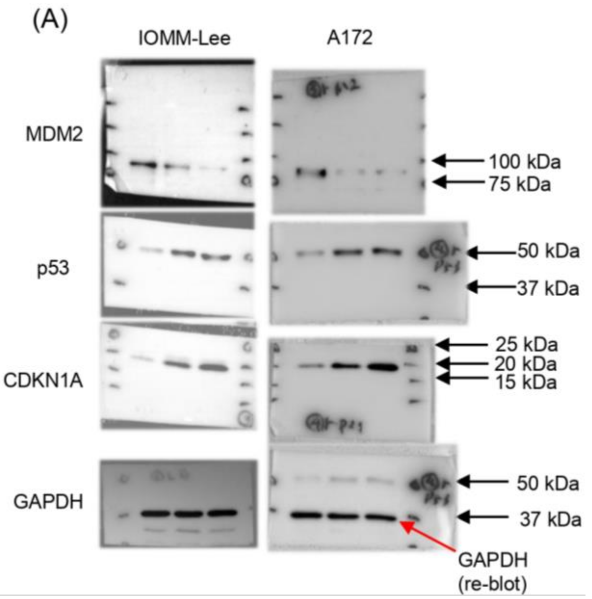

Figure S2. Uncropped Western Blot images for Figure 2.

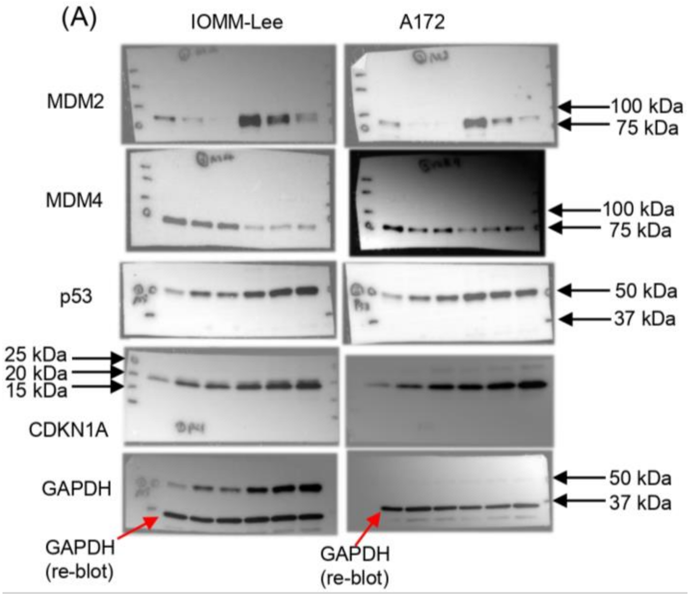

Figure S3. Uncropped Western Blot images for Figure 3.

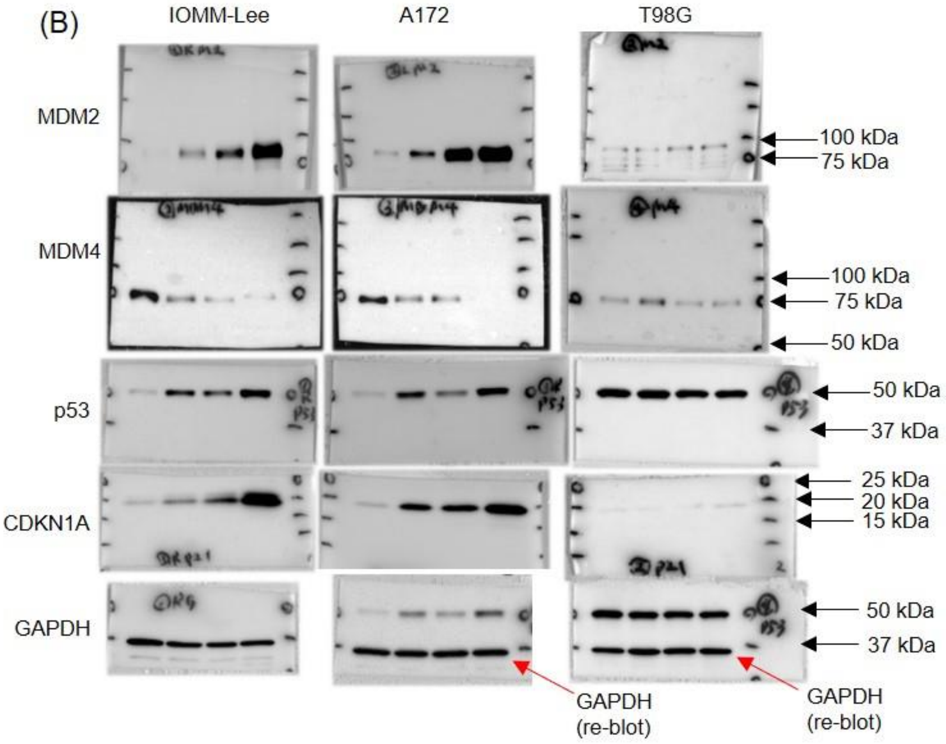

Figure S4. Uncropped Western Blot images for Figure 4.
